# Recombination shapes African swine fever virus serotype-specific locus evolution

**DOI:** 10.1038/s41598-020-75377-y

**Published:** 2020-10-28

**Authors:** Mariia Nefedeva, Ilya Titov, Sodnom Tsybanov, Alexander Malogolovkin

**Affiliations:** Federal Research Center for Virology and Microbiology, Volginsky, Russia

**Keywords:** Virology, Computational biology and bioinformatics, Evolution

## Abstract

The recombination is one of the most frequently identified drivers of double-stranded DNA viruses evolution. However, the recombination events in African swine fever virus (ASFV) genomes have been poorly annotated. We hypothesize that the genetic determinants of ASFV variability are potential hot-spots for recombination. Here, we analyzed ASFV serotype-specific locus (C-type lectin (*EP153R*) and CD2v (*EP402R*)) in order to allocate the recombination breakpoints in these immunologically important proteins and reveal driving forces of virus evolution. The recombinations were found in both proteins, mostly among ASFV strains from East Africa, where multiple virus transmission cycles are notified. The recombination events were essentially associated with the domain organization of proteins. The phylogenetic analysis demonstrated the lack of clonal evolution for African strains which conclusively support the significance of recombinations in the serotype-specific locus. In addition, the signature of adaptive evolution of these two genes, *p*N/*p*S > 1, was demonstrated. These results have implications for the interpretation of cross-protection potential between evolutionary distant ASFV strains and strongly suggest that C-type lectin and CD2v may experience substantial selective pressure than previously thought.

## Introduction

African swine fever virus (ASFV) is a DNA virus of *Asfarviridae* family that causes a contagious and fatal disease of domestic pigs and wild boars, and is characterized by fever, skin cyanosis and extensive haemorrhages in organs. Mortality in infected herds may approach up to 100%^1,2^. The African swine fever virus genome is a single linear double-stranded DNA (dsDNA) molecule between 170 and 190 kbp in size, depending on the isolate and encoding approximately 165 viral proteins^3,4^. The DNA has covalently closed ends with terminal inverted repeats (TIR) with the array of different tandem repeats adjacent to the termini^5,6^. Twenty-four ASFV genotypes (I–XXIV) based on the nucleotide sequences variation in the C-terminal region of the *B646L* gene, encoding the major capsid protein p72, were identified^7^. Alternative genetic typing using *EP402R* gene, encoding the CD2v protein, provides better discrimination of biologically pertinent phenotypes. Our recent finding also shows that the ASFV CD2v protein demonstrates clear correlation with hemadsorption inhibition assay (HAI), and is significant for cross-protection^8^. By swapping serotype-specific locus (*EP153R* and *EP402R*) between heterologous ASFV strains, we demonstrated the impact of this change on the disease severity, viremia and its protective potential. In a follow-up study, we additionally demonstrated that C-type lectin and CD2v have 6 clusters of functional T-cell epitopes which are undoubtedly important elements of cross-protective immunity^9^. We highlighted that both ASFV C-type lectin/*EP153R* and CD2v/*EP402R* are important for serotype-specific protection but cannot provide an ultimate protective immunity without other equally important virus antigens. It’s worth mentioning that the T-cell epitopes of the CD2v were also found by other research group^10^. Intriguingly, p72 and CD2v-based phylogenetic analysis clustered identical ASFV isolates in different unrelated groups, prompting that recombination events might occur in ASFV genome.

Recombination is an important driving force of evolution in many viral families (i.e. *Flaviviridae*, *Bromoviridae*, *Coronaviridae* etc.) having the potential to combine favorable mutations into one genome^11^. Recombination events usually occur between species with high sequence similarity (e.g., homologous viruses)^12,13^. The recombination process is well known for DNA viruses, such as herpesviruses and adenoviruses, which recombine with closely related viruses^14^. The recombinations have also been observed among many other DNA virus families, including poxviruses, geminiviruses and anelloviruses^15,16^. However, it is worth noting that the identification of the recombination events based on the limited virus genomic sequences is not a trivial task^17,18^. There is little data available about the recombinations among the ASFV genomes. Nonetheless, some reports assume that the recombination breakpoints may be present in several ASFV genes^2^.The study of p54 (*E183L*) sequences of sixteen ASFV strains revealed several recombination events in the ASFV strains isolated in Italy and one isolate from South Africa^19^. Similar analysis of the *B646L* and *CP204L* genes demonstrated the absence of recombination events, non-synonymous bias and a small number of codons under positive selection, which may indicate the natural virus evolution^19^.Computational analysis of available ASFV genomes reveal multiple reticulate events in virus phylogeny^20,21^.

The ASFV has a unique fitness for DNA viruses and is capable of efficient replication in ticks (*Ornithodoros*) and pigs. However, the exact host-range determinants of the virus have not been identified yet. Nevertheless, several ASFV proteins demonstrate peculiar host specificity. One of these, ASFV CD2v protein, has been shown to be an essential for virus replication in ticks, but not in pigs^22^. Significant homology between virus CD2v protein and mammalian T-cell adhesion molecules (CD2), as well as among C-type lectin proteins, may serve a perfect basis for homologous recombination. Furthermore, mixed infection of pigs with genetically different ASFV strains has been demonstrated in East African countries^23,24^. Infection with two and more strains, potentially, may lead to the creation of recombinant virus with a patchy genome.

Taking together, we hypothesize that serotype-specific locus consist of C-type lectin and CD2v, might be a hot-spot for drastic evolutionary changes due to: (i) unprecedented selective pressure during interspecies transmission between ticks and pigs; (ii) significant homology between host and virus genes; (iii) striking difference between CD2v/C-type lectin and p72 phylogeny; (iv) noticed mixed infection in pigs with multiple ASFV strains.

The aim of this work is to identify the recombination patterns in ASFV serotype-specific proteins using an array of bioinformatics methods and understand the significance of recombination for virus fitness.

Here, we focused on the comparative analysis of the ASFV sequences of two proteins *EP153R* (C-type lectin) and *EP402R* (CD2v), which we believe are important components for multimodal protective immunity against ASFV. The controversial results regarding the significance of *EP153R* (C-type lectin) and *EP402R* (CD2v) in cross-protection reported for different ASFV strains^8,10,25^ set a logical basis for the current study. Despite the decades of work on *EP402R*/CD2v and *EP153R/*C-type lectin little do we know about the physical interactions of those two proteins, their molecular structures and evolution. The unique characteristics of those two proteins provide valuable information about the breadth of ASFV genomes, antigenic variations, and high resolution phylogenetic reconstructions.

In our manuscript, we used an arsenal of bioinformatic methods to demonstrate the significance of the reticulate events (i.e. recombination) on virus evolution in general and serotype-specific locus in particular. We used a recombination analysis, population genetics (dS/dN), we measured the direct effect of natural selection by polymorphism analysis (pS/pN), we detected amino acids sites under positive selection by fixed effect likelihood analysis (FEL), we computed the phylogenetic networks and performed pairwise genetic identity calculations. Altogether, we postulate that our finding can undoubtedly infer that the C-type lectin/*EP153R* and CD2v/EP402R are the subjects for the recombination.

We found a statistically significant evidence of recombinations in the AFSV C-type lectin and CD2v proteins. The results of serotype-specific proteins bioinformatic analysis indicate the peculiar evolution of those proteins and its implication to cross-protection between distant ASFV strains.

## Results

### Recombinations among the ASFV C-type lectin and CD2v proteins

The nucleotide sequences of the ASFV C-type lectin and CD2v available at the GenBank were used for the recombination analysis. After primary filtration and quality assessment seventy paired sequences were selected. The nucleotide sequences were translated to amino acids and subsequently back translated to the nucleotides using PAL2NAL server. Therefore, the ASFV C-type lectin and CD2v codon-based alignments were created and used for following analysis. The detection methods implemented in the RDP4 software confidently identified the recombination events in the ASFV C-type lectin and CD2v proteins. In detail, among seventy ASFV strains five recombinants were identified in C-type lectin sequences and five in CD2v with strong statistical significance (Table 1).

**Table 1:** The ASFV strains with identified recombination events in C-type lectin (*EP153R*) and CD2v (*EP402R*).

ASFV protein/gene	ASFV strain (GeneBank number)	Detection method^a^	p-value^b^	Position
C-type lectin (*EP153R*)	Kenya 05/TK1 (KM111294)	R G B M C S T	1.54 × 10^–13^	192–462
	Tengani-1962 (AY261364)	G B M C S T	1.5 × 10^–11^	192–462
	Malawi-Lil (AY261361)	G B M C S T	4.48 × 10^–11^	28–220
	Kenya-1950 (AY261360)	G B M C S	1.37 × 10^–9^	15–246
	TSP-80 (KM609359)	G B M S	2.16 × 10^–6^	57–232
CD2v (*EP402R*)	Rhodesia (KM609354)	R G B M C S T	8.17 × 10^–14^	526–673
	Tengani-1962 (AY261364)	R G B M C S T	3.26 × 10^–10^	316–633
	Warthog (AY261366)	R G B M C S T	9.68 × 10^–10^	801–1017
	Uganda (KM609361)	R G B M C S T	6.04 × 10^–8^	790–1072
	Spencer (KM609357)	R G B M C T	2.44 × 10^–5^	29–932

^a^Detection methods implemented in RDP4: *R* RDP, *G* GENECON, *B* Bootscan, *M* Max-Chi, *C* Chimaera, *S* SiScan, *T* TOPALDSS.

^b^Bootscan p-values supporting the recombination breakpoints.

The ASFV strains from East Africa (Kenya, Malawi, Uganda, Tanzania, Zimbabwe) formed the majority of recombinant strains. No evidence of recombination was found among the recent ASFV strains (Genotype II, Serogroup 8) causing epidemics in Western and Eastern Europe, Russia, and South-East Asia. The absence of recombinations among those strains suggests that the clonal expansion of the virus population took place from a single virus pool. Intriguingly, the recombination pattern in the ASFV CD2v protein was detected in the ancestor ASFV strain Rhodesia (KM609354), originated from Zimbabwe in the early eighties. The recombination breakpoints were found around the transmembrane region of the ASFV Rhodesia CD2v protein. Similar pattern (clonal expansion) was discovered for the historical ASFV strains isolated in Europe between 1960 and 1995, belonging to Genotype I, Serogroup 4.

**Figure 1:**
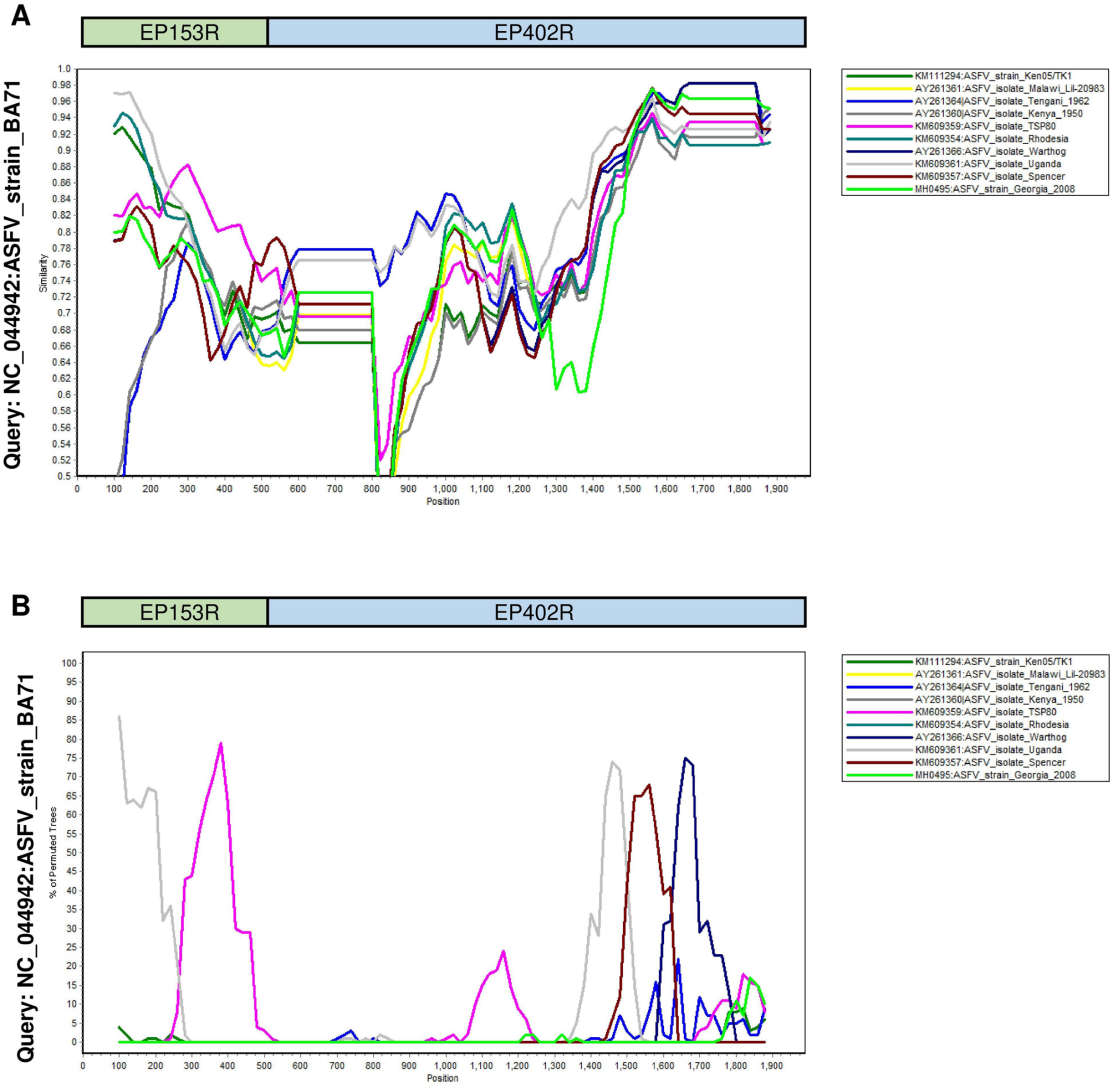
Similarity plot (**A**) and Bootscan analysis (**B**) of the concatenated *EP153R/EP402R* genes from identified recombinant ASFV strains. The analysis was performed against reference ASFV BA71 strain (GenBank Acc. Number NC_044942) as a query which was classified as a non-recombinant strain. Similarity plots demonstrate the sequence similarity between the query sequence and the other sequences. Bootscan analysis shows fragmented pattern as a result of recombination^26^.

The ASFV C-type lectin (*EP153R*) and CD2v (*EP402R*) sequences from recombinant ASFV strains were concatenated and similarity and bootscan plots were produced (Fig. 1). The bootscan analysis indicates the fragmented pattern of the ASFV C-type lectin (*EP153R*) and CD2v (*EP402R*) as a result of the recombination.

To demonstrate additional evidence against the null hypothesis, several ASFV structural proteins (p72 (*B646L*) and p30 (*CP204L*)) were added to the recombination analysis using the same algorithm. The results indisputably confirmed the absence of any recombination events in those proteins. The data from the recombination analysis demonstrate the presence of gene rearrangements in the ASFV serotype-specific proteins.

### Population dynamics, polymorphisms and variability of C-type lectin and CD2v proteins

The ASFV C-type lectin (*EP153R*) and CD2v (*EP402R*) are among the most variable genes in the virus genome. Having exceptional variability and increased number of publicly available ASFV sequences, the population dynamics analysis is of interest for understanding the serotype-specific locus evolution. Evolutionary pressures on these two proteins were initially quantified by the ratio of nonsynonymous (dN) to synonymous (dS) mutation rates. The dN/dS ratio for C-type lectin and CD2v was 0.5 and 0.46, respectively (Table 2). The dN/dS < 1 suggests that natural selection suppresses the changes in those proteins and foster purifying selection. Despite its simplicity and robustness, dN/dS analysis is less sensitive for protein-coding sequences from a single population (species) than divergent population (different species)^27^.

A viable alternative for understanding the selective pressure within a certain population is a polymorphism analysis. The ratio of non-neutral polymorphisms (pN) to neutral (pS) for C-type lectin and CD2v proteins was calculated using McDonald-Kreitman test^28^ and was 2.04 and 3.17, respectively. The pN/pS ratio > 1 demonstrates that two proteins experience strong selection pressure and natural selection promotes changes in those proteins.

The ASFV C-type lectin and CD2v proteins are transmembrane glycoproteins having intracellular and extracellular domains. Thus, it is highly likely that some regions of those proteins may experience different selective pressures. The Fixed Evolutionary Likelihood analysis (FEL) revealed several amino acids which experience strong selective pressure. Five amino acids were identified for C-type lectin protein and two for CD2v protein. The diversifying sites identified in C-type lectin and CD2v summarized in Table 2.

**Table 2:** The ratio of divergence at nonsynonymous and synonymous sites (*d*S/*d*N) and relative abundance of non-synonymous and synonymous polymorphisms (pN/pS) in the ASFV the C-type lectin and CD2v proteins and the sites under diversifying/pervasive selection.

ASFV proteins	dN	dS	ω	Pn	Ps	pN/pS	Amino acids under positive selection*
C-type lectin	0.17	0.34	0.5	47	23	2.04	9 (G) 14 (N) 23 (S) 98 (R) 153 (K)
CD2v	0.18	0.39	0.46	146	46	3.17	252 (E) 359 (P)

*dN* non-synonymous mutation substitution rate, *dS* synonymous mutations substitution rate, *ω* dN/ds ratio, *pN* number of non-neutral polymorphism, *pS* number of neutral polymorphism.

*Positive diversifying selection sites identified by the Fixed Evolution likelihood (FEL) with p-value < 0.05. The amino acids under selective pressure are highlighted in bold. The affected are presented in brackets. Positive diversifying selection sites identified by the Fixed Evolution likelihood (FEL) with p-value < 0.05. The amino acids under selective pressure are shown in brackets. The codon positions are given according to the ASFV BA71 reference strain (GenBank accession number NC_044942).

The results of full FEL analysis with negative and positive selection sites for the ASFV C-type lectin (*EP153R*) and CD2v (*EP402R*) genes are available in supplementary materials (S1 and S2). The ASF C-type lectin and CD2v protein sequence alignments with identified codons under selective pressure can be found in Supplementary Materials (S3 and S4).

**Figure 2:**
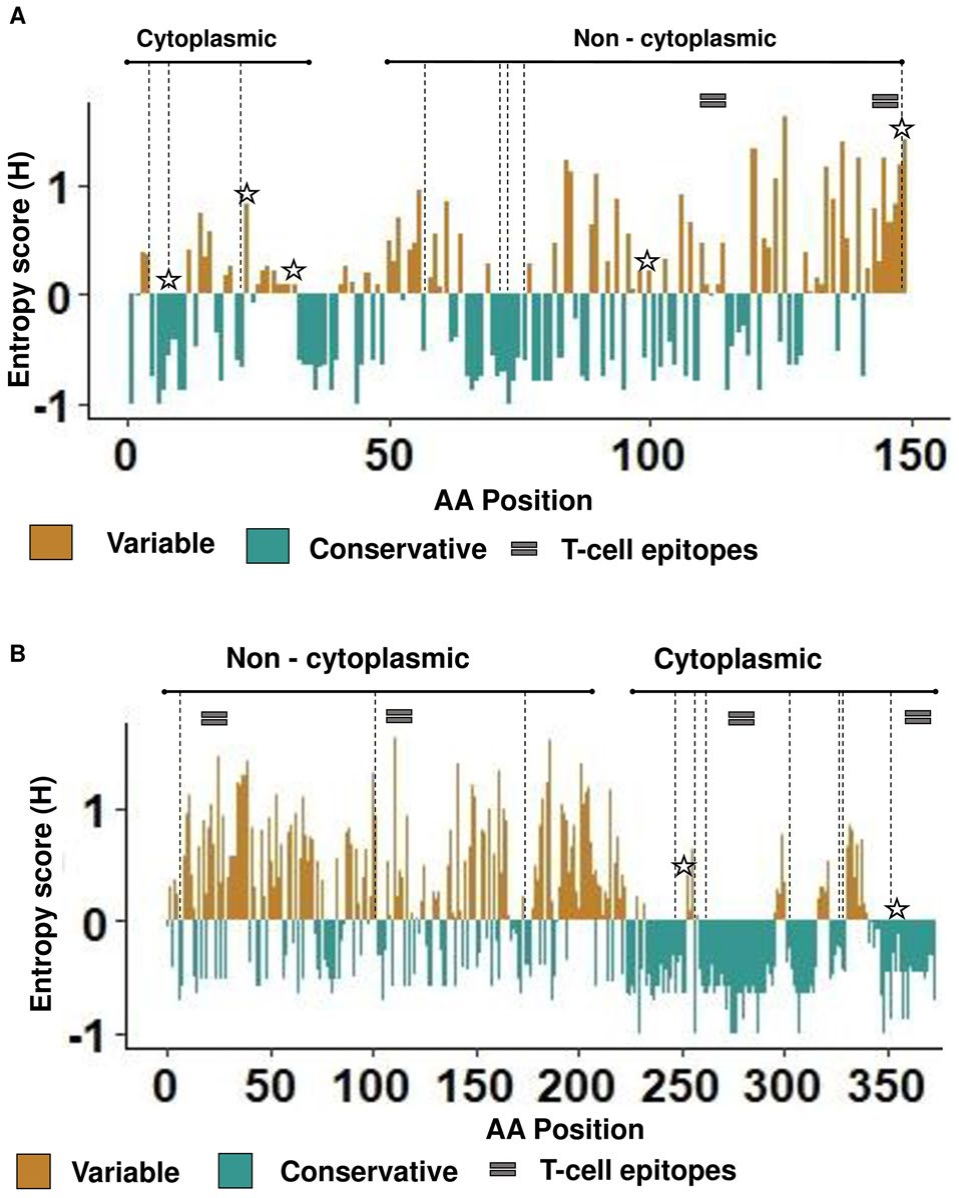
Analysis of ASFV C-type lectin (**A**) and CD2v (**B**) proteins variability, recombinations and sites under selective pressure. Conservative amino acids highlighted in green, variable amino acids are in light brown. The recombination breakpoints are marked by dash line. Amino acids under positive selection depicted as stars. Experimentally identified T-cells epitopes represented by grey rectangles.

In addition, the protein variability analysis was used to determine the most variable and conservative sites among the C-type lectin and CD2v sequences. The protein alignments were analyzed using Shannon entropy test as the most sensitive tool to detect the diversity within the population. The variable sites were mapped on the C-type lectin and CD2v sequence together with previously predicted/ identified T-cell epitopes, recombination breakpoints and the sites under diversifying selection (Fig. 2).

The results show that the extracellular domains are the most variable parts of the proteins. Interestingly, the recombination breakpoints have been identified mostly in the variable regions spanning the conservative parts. The amino acids under positive selection were found in conservative and variable sites.

### Phylogenetic network of the C-type lectin and CD2v proteins

The recombination events dramatically shape the evolution of the species and leave footprints in phylogenetic relationships. The recombinant species have mosaic genes/genomes and stand out in the phylogenetic tree as intermediate variants between two parental species. Due to the multiple and sometimes convoluted nature of evolutionary mechanisms (e.g. gene transfer, hybridization and recombination), widely-used phylogenetic models for phylogenetic trees reconstruction may miss important evolutionary signals and lead to the misinterpretation of phylogenetic relationship. This becomes even more essential for the phylogenetic analysis of extremely variable sequences, such as C-type lectin/*EP153R* and CD2v/*EP402R.* In addition, in case of reticulate evolutionary events, no tree-based model can accurately model the data.

**Figure 3:**
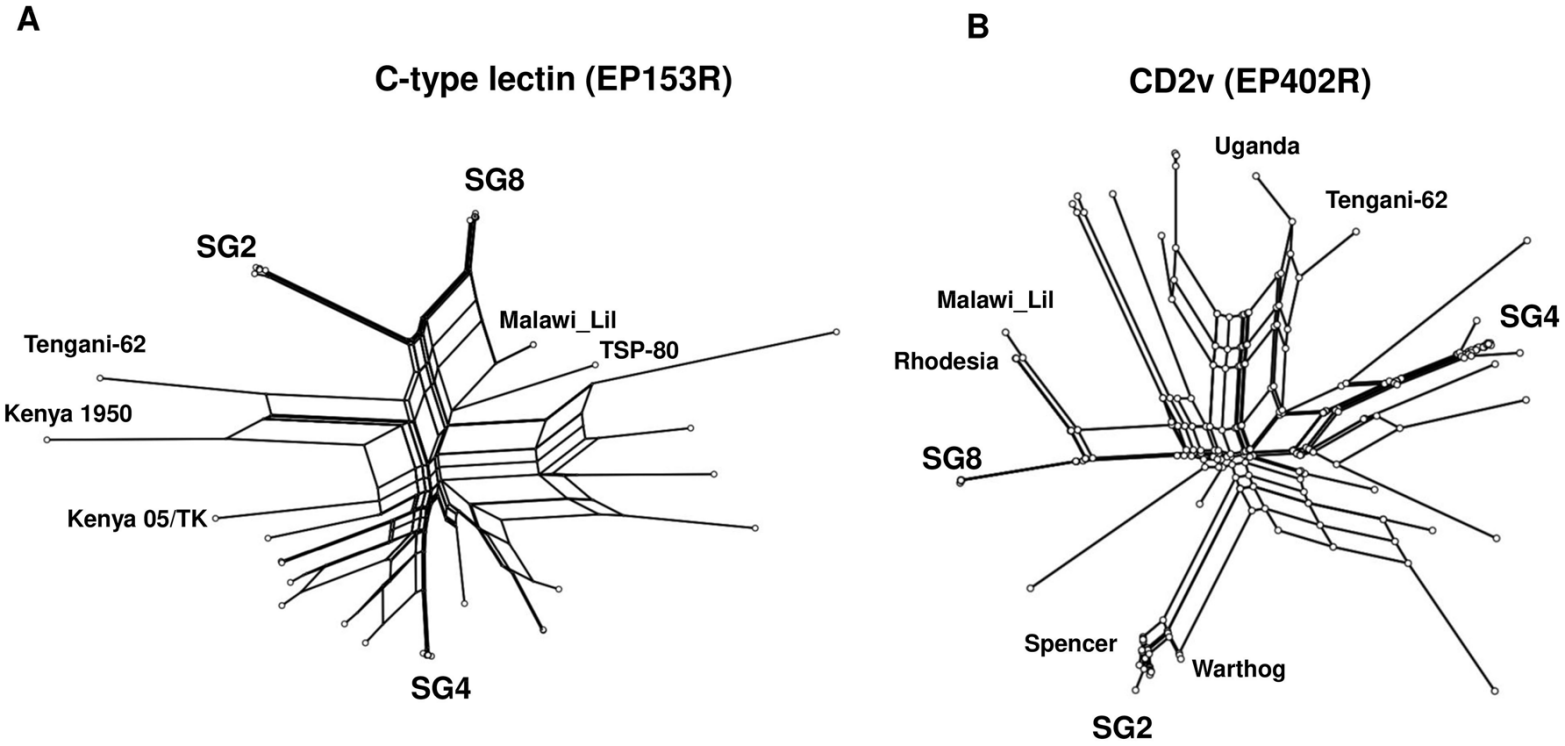
Phylogenetic network analysis of ASFV C-type lectin (**A**) and CD2v (**B**) proteins. Recombinant ASFV strains identified by the RDP4 assigned in the network. ASFV serogroups (SG2, SG4 and SG8) forming monophyletic clusters are allocated. The phylogenetic networks were produced using SplitsTree5^29^.

We performed the phylogenetic network analysis using generated codon-based alignments of C-type lectin and CD2v proteins. We chose the split networks to represent the confidence sets of trees for whether the conflicting signal in a network is treelike, where a phylogenetic tree corresponds to a collection of compatible splits. Despite the unfamiliar structure of phylogenetic network, it is very similar to the phylogenetic tree topology. Briefly, taxa in phylogenetic network are represented by nodes and evolutionary relationships are depicted by edges. Any reticulate event has a dramatic effect on the phylogenetic relationship between the taxa and result in the reticulation nodes. Phylogenetic network of both proteins showed extreme diversity and heterogeneity (Fig. 3). The ASFV strains isolated during the massive epidemics formed the uniform clusters and demonstrated monophyletic topology of the tree. Thus, the modern ASFV isolates causing disease outbreaks in Europe, Russia and South-East Asia were grouped together. The similar clonal expansion pattern was found for the historical European ASFV isolates belonging to the Genotype I, Serogroup 4. Additional cluster was discovered, among the ASFV isolates belonging to various genotypes, but Serogroup 2 only.

**Figure 4:**
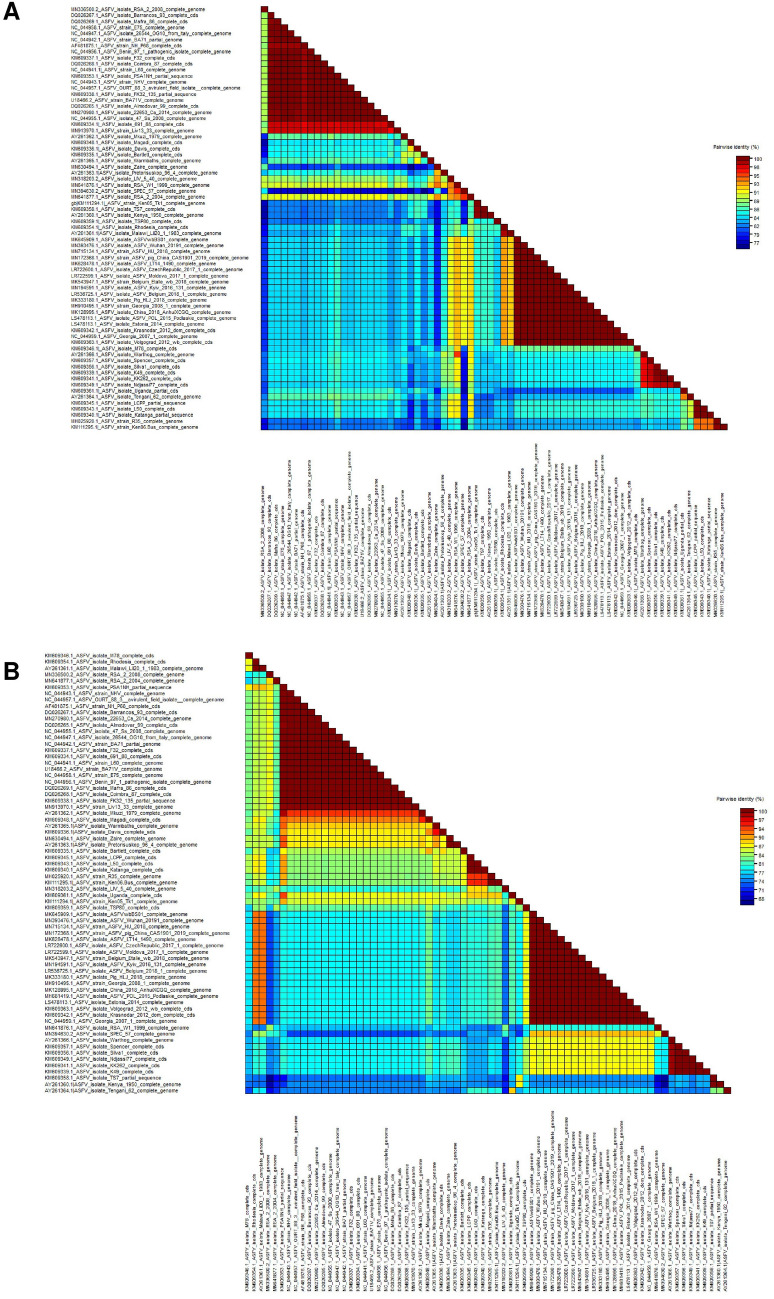
Pairwise genetic identity matrices of ASFV C-type lectin (**A**) and CD2v (**B**) proteins. The color indicates the homology level between the sequences. Recombinant ASFV strains have the lowest homology among the analyzed strains. The pairwise matrices were generated using SDT v.1.2^30^.

In contrast, the recombinant ASFV strains had a distinguished location in the network and didn’t group with any strains. Therefore, the phylogenetic evidence also supports the presence of recombinations in the ASFV C-type lectin and CD2v proteins.The pairwise identity matrix additionally demonstrated that the recombinant ASFV strains have limited homology with other strains (Fig. 4).

The results of the sequence demarcation tool highlighted the same homologous clusters that were identified in the phylogenetic network analysis. The sequence demarcation analysis is a great tool allowing easy visualization of homology between sequences. Interestingly, the ASFV Rhodesia stain showed high homology to the modern ASFV isolates as well as ASFV Malawi strain pointing to the Serogroup 8 cluster.

## Discussion

Population dynamics of DNA viruses is different from their RNA acquaintances. The mutation rate among the DNA viruses is significantly lower than for error-prone RNA viruses^31,32^. Nevertheless, the selective pressure created by the host immune system equally shapes the evolution of DNA viruses and pushes them to increase their fitness over and over again. The arboviruses are constantly under the crossfire between two immune systems: insect vector and mammalian host. The unique biological cycle of arboviruses indisputably leaves the marks in the virus genome and is of interest for understanding the mechanism of virus adaptation and evolution. The recombination events have been found in many arboviruses and we believe that the ASFV is not an exception. However, no thorough recombination analysis has been done so far due to the limited number of sequences available.

The ASFV is a unique DNA arbovirus which infects ticks and pigs, and host-range determinants are encoded in the virus genome, but remains to be discovered^33^. We and others have previously noticed unprecedented incongruence in phylogeny between the ASFV genes^2,19^. The phylogenetic analysis of the ASFV major capsid protein p72 (*B646L*) and C-type lectin and CD2v demonstrate remarkable difference in tree topology, pointing at potential recombination in ASF genomes. Taking into account, the role of CD2v protein in virus dissemination after infection via red blood cell transfer and significance for replication in ticks, we hypothesize that this protein may accumulate the signatures of positive selection over divergent lineages and worth considering for recombination analysis.

Deciphering the role of recombination events in serotype-specific locus of ASFV has important implications toward the understanding the cross-protective potential between genetically distant virus strains.

The identification of recombinant ASFV strains in East Africa underlines the complexity of ASFV biology in this region^34^. Long history of multiple ASFV genotypes circulating in this region, with a variety of indigenous species (domesticated pigs, warthogs, bush pigs, giant forest hogs) and vectors (*Ornithodoros* ticks) involved in transmission cycle make the region a perfect “mixing bowl” for the recombination. The recombinant viruses with the increased fitness may spread like a wildfire within the susceptible population and have a great potential for transboundary transmission among the naive populations.

The recombination breakpoints in C-type lectin and CD2v proteins were found in intracellular and extracellular domains. We hypothesize that the proteins domains are the fixed structural units and the location of the breakpoints is likely associated with the B- and T-cell epitopes. Despite the several computational attempts to predict the ASFV B- and T-cell epitopes^9,35,36^, limited experimental data are available to make a decisive conclusion. The understanding of the cross-protective potential of recombinant ASFV strains may shed a light on the role of recombination in ASFV serotype-specificity. Several recombination breakpoints spanning transmembrane region were also identified in the ASFV Rhodesia, Malawi-Lil and TSP-80 strains. The membrane proteins from other species are capable to evolve using diverse mechanisms, including recombination^37^. The “jumping” replication cycle of ASFV (from ticks to pigs) may facilitate the virus evolution and explain the mosaic characteristics around the transmembrane region. Additional ASFV sequences isolated from ticks will undoubtedly help to understand the origin of C-type lectin and CD2v genes and evolutionary mechanism driving virus fitness.

The ASFV C-type lectin and CD2v are the extremely variable proteins and were successfully used to improve the resolution of the phylogenetic analysis and genotyping^38,39^. Being membrane proteins, both proteins experience a selective pressure and have a hallmark of diversifying selection. The dN/dS ratio for population dynamics of homologous sequences demonstrated relatively low values and confirmed to be insensitive^27^. In contrast, the polymorphism ratio showed clear evidence of promoted changes and natural selection in both proteins. As a follow up study, the population dynamics of the ASFV strains from ticks would be absolutely necessary for precise characterization of virus evolution in different hosts.

The specific sites under positive selective pressure were identified in serotype-specific proteins of the ASFV using FEL analysis. These sites are important indicators of protein evolution and should be considered for C-type lectin and/or CD2v proteins-based vaccine design. Importantly, the mutations in these sites may dramatically change the architecture of the proteins and as a result its functionality. Unfortunately, the structure of the ASFV C-type lectin and CD2v is still unknown and structural modelling of the compensatory mutations is not possible yet.

At the time of writing the manuscript, essential orthogonal proofs of diversifying selection in ASFV *EP153R*/C-type lectin and *EP402R*/CD2v has been released as a preprint by Bao et al.^21^. Using newly developed tool called “Sweep Cluster” and other alternative computational methods, the authors confirmed our finding of decent selective pressure on the ASFV C-type lectin and CD2v proteins. These compelling evidence emphasize the significance of our results and the importance of serotype-specific locus in ASF immunity^21^. Overall, this finding suggests that ASFV serotype-specific proteins (C-type lectin and CD2v) are known to evolve by homologous recombination and experience strong selective pressure. Nevertheless, the recombination is not the sole driver of ASFV evolution and many aspects of virus fitness are remain to be found.

## Methods

### Sequences

The 70 nucleotide sequences of two ASFV genes *EP153R* (C-type lectin) and *EP402R* (CD2v) were used in all analysis and are available in S4.

### Recombination analysis

The PAL2NAL server was used for multiple codon alignment and converting the nucleotide sequences into amino acids and back translation to the nucleotides^40^. The Mega 5 software was used for a nucleotide multiple alignment by using MUSCLE^41,42^. Identification of recombination breakpoints was carried out with RDP4 software, which contains an extensive array of algorithms for detecting recombinations^11,43–48^. Nucleotide sequences of ASFV *B646L* (p72), and *CP204L* (p30) genes were used as a threshold control of recombination. A putative recombination breakpoints were retained to subsequent analysis only when it was consistently identified by at least four algorithms available in RDP4.

The ASFV genes *EP153R* (C-type lectin) and *EP402R* (CD2v) from newly identified recombinant strains were concatenated and analyzed by Bootscan (window 200 bp, step 20 bp, replicates 1000, Kimura 2-paramenter model and Neighbor-joining algorithm) implemented in SimPlot (v. 3.5.1).

### *d*S/*d*N ratio and *p*N/*p*S ratio

To determine the number of synonymous (synonymous distance, *d*S) and nonsynonymous substitutions (nonsynonymous distance, d*N*) per site the ASFV C-type lectin (*EP153R*) and CD2v (*EP402R)* proteins, as well as the number of potentially synonymous and nonsynonymous sites for each codon based on the equal frequencies hypothesis of all nucleotide substitutions there were analysed with CodeML implemented in PAML (Phylogenetic Analysis by Maximum Likelihood)^49^. Definition of the ratio of non-neutral polymorphisms (*p*N) to neutral (*p*S) for C-type lectin and CD2v proteins for measure the direct effect of natural selection removing slightly deleterious non-synonymous variants was calculated using McDonald-Kreitman test^28^.

### Detection of evolutionary selection across individual sites

Datamonkey web-server was used for detecting sites under selection by Fixed effects likelihood (FEL) method used for identifying sites that may have experienced pervasive diversifying or purifying selection^50,51^.

### Phylogenetic networks

For computing phylogenetic networks for codon-based alignments of C-type lectin and CD2v proteins SplitsTree5 was used^29^.

### SDT analysis

Pairwise genetic identity calculations was performed to visualize the homology among the ASF *EP153R* and *EP402R* genes using SDT v1.2 software (Sequence Demarcation Tool)^30^.

### Ethical approval

An ethical review was not required for this study, according to local and national legislation, because this work does not contain any experiments on animals.

## Supplementary information

Supplementary Legends.

Supplementary Dataset.

Supplementary Table S1.

Supplementary Table S2.

Supplementary Table S3.

Supplementary Table S4.
