# Supplementary material for: Recombination shapes African swine fever virus serotype-specific locus evolution
Source: Sci Rep. 2020 Oct 28;10:18474. doi: 10.1038/s41598-020-75377-y (PMC7794389; doi:10.1038/s41598-020-75377-y)
Supplement: Supplementary file 1 — Supplementary Legends. [file 41598_2020_75377_MOESM1_ESM.docx]

**Supplementary information** for the manuscript

**Title:** Recombination Shapes African Swine Fever Virus Serotype-Specific Locus Evolution

**Authors:** Mariia Nefedeva^1^, Ilya Titov^1^, Sodnom Tsybanov^1^, and Alexander Malogolovkin^1,2^

**Affiliation:**^1^Federal Research Center for Virology and Microbiology, Volginsky, Russia

^2^London School of Hygiene and Tropical Medicine, Keppel Street, London, WC1E 7HT, UK

***Corresponding author:**

Alexander Malogolovkin, DVM, MSc, PhD. E-mail: alex.malogolovkin@lshtm.ac.uk

**Journal name:** Scientific Reports

***Content***

**Supplementary material S1.**The results of full FEL analysis with negative (dark gray) and positive (green) selection sites identified in C-type lectin protein (*EP153R*).

**Site** - Site Position; **Partition** - Partition that site belong to; **alpha** - Synonymous substitution rate at a site; **beta** - Non-synonymous substitution rate at a site; **omega** - Ratio of nonsynonymous to synonymous substitution rate; **alpha=beta** - The rate estimate under the neutral model; **LRT** - Likelihood ration test statistic for beta = alpha, versus beta &neq, alpha; **p-value** - the p-value threshold to use when testing for selection; **Total branch length** - The total length of branches contributing to inference at this site, and used to scale *dN/dS.*

**Supplementary material S2.**The results of full FEL analysis with negative (dark gray) and positive (green) selection sites identified in CD2v protein (*EP402R).*

**Site** - Site Position; **Partition** - Partition that site belong to; **alpha** - Synonymous substitution rate at a site; **beta** - Non-synonymous substitution rate at a site; **omega** - Ratio of nonsynonymous to synonymous substitution rate; **alpha=beta** - The rate estimate under the neutral model; **LRT** - Likelihood ration test statistic for beta = alpha, versus beta &neq, alpha; **p-value** - the p-value threshold to use when testing for selection; **Total branch length** - The total length of branches contributing to inference at this site, and used to scale *dN/dS*.

**Supplementary material S3.** The protein alignments of ASFV C-type lectin (*EP153R*). The amino acids under the selective pressure according to the FEL analysis are marked by asterisk (A). The protein alignments of ASFV CD2v (*EP402R*). The amino acids under the selective pressure according to the FEL analysis are marked by asterisk (В). The alignments were produced by UGENE v35.1 [52].

**Supplementary material S4**.The dataset of the ASFV *EP153R* (C-type lectin) and *EP402R* (CD2v) genes used in this study with the GenBank accession numbers and assigned genotypes.
