# Supplementary material for: Recombination shapes African swine fever virus serotype-specific locus evolution
Source: Sci Rep. 2020 Oct 28;10:18474. doi: 10.1038/s41598-020-75377-y (PMC7794389; doi:10.1038/s41598-020-75377-y)
Supplement: Supplementary file 2 — Supplementary Dataset. [file 41598_2020_75377_MOESM2_ESM.pdf]

Supplementary material for the manuscript

Title: Recombination Shapes African Swine Fever Virus Serotype-Specific Locus Evolution

Authors: Mariia Nefedeva[1], Ilya Titov[1], Sodnom Tsybanov[1], and Alexander Malogolovkin[1,2*]

Affiliation:[1]Federal Research Center for Virology and Microbiology, Volginsky, Russia

[2]London School of Hygiene and Tropical Medicine, Keppel Street, London, WC1E 7HT, UK

***Corresponding author:**

Alexander Malogolovkin, DVM, MSc, PhD. E-mail: alex.malogolovkin@lshtm.ac.uk

Journal name: Scientific Reports

Content

Supplementary material S1. The results of full FEL analysis with negative (dark gray) and positive (green) selection sites identified in C-type lectin protein (*EP153R*).

Site - Site Position; **Partition** - Partition that site belong to; **alpha** - Synonymous substitution rate at a site; **beta** - Non-synonymous substitution rate at a site; **omega** - Ratio of nonsynonymous to synonymous substitution rate; **alpha=beta** - The rate estimate under the neutral model; **LRT** - Likelihood ration test statistic for beta = alpha, versus beta & neq, alpha; **p-value** - the p-value threshold to use when testing for selection; **Total branch length** - The total length of branches contributing to inference at this site, and used to scale dN-dS.

Site	Partition	alpha	beta	omega	alpha=beta	LRT	p-value	Total branch length
1	1	0	0	NaN	0	0	1	0
2	1	0	0,412	Infinity	0,334	1,092	0,296	17,101
3	1	107,669	0,947	0,009	0,965	2,982	0,084	949,848
4	1	0,278	0,684	2,457	0,498	0,886	0,347	30,69
5	1	0	0,066	Infinity	0,04	1,026	0,311	2,723
6	1	0,367	0,04	0,109	0,129	2,921	0,087	4,758
7	1	0,216	0,041	0,191	0,111	1,624	0,202	3,535
8	1	0,493	0	0	0,278	7,067	0,008	4,166
9	1	0	0,556	Infinity	0,218	6,707	0,01	23,046
10	1	0	0,059	Infinity	0,023	1,155	0,282	2,438
11	1	0,517	0,038	0,074	0,248	5,846	0,016	5,956
12	1	13,91	0,172	0,012	0,579	13,222	0	124,757
13	1	0,378	0,339	0,897	0,354	0,013	0,91	17,248
14	1	0	0,796	Infinity	0,388	6,1	0,014	33,006
15	1	0,242	0,279	1,153	0,274	0,002	0,968	13,602
16	1	3,45	0,305	0,088	0,688	6,346	0,012	41,82
17	1	2,43	0,226	0,093	0,537	4,861	0,027	29,928
18	1	0,375	0,1	0,267	0,125	0,418	0,518	7,315
19	1	0	0,068	Infinity	0,058	1,027	0,311	2,812
20	1	0	0,22	Infinity	0,172	0,869	0,351	9,108
21	1	0,72	0,336	0,468	0,379	0,186	0,666	20,034
22	1	0	0,162	Infinity	0,146	0,576	0,448	6,732
23	1	0	0,655	Infinity	0,409	5,228	0,022	27,153
24	1	0	0,629	Infinity	0,509	2,618	0,106	26,078
25	1	0,252	0	0	0,069	2,914	0,088	2,128
26	1	0	0,2	Infinity	0,104	0,518	0,472	8,312
27	1	0,437	0,367	0,838	0,385	0,021	0,885	18,898
28	1	0,726	0,1	0,138	0,348	3,898	0,048	10,293
29	1	0,361	0,194	0,538	0,218	0,154	0,695	11,102

30	1	0,673	0,6	0,892	0,631	0,018	0,895	30,564
31	1	0,114	0,013	0,116	0,058	0,898	0,343	1,517
32	1	0,201	0	0	0	-0,292	1	1,703
33	1	0,282	0,343	1,217	0,32	0,035	0,852	16,589
34	1	0,235	0	0	0,099	3,036	0,081	1,985
35	1	0,845	0,099	0,118	0,304	3,734	0,053	11,271
36	1	0,88	0	0	0,297	7,497	0,006	7,442
37	1	0,882	0,195	0,221	0,432	2,929	0,087	15,537
38	1	0	0	NaN	0	0	1	0
39	1	0,617	0	0	0,182	8,066	0,005	5,218
40	1	0,291	0,325	1,116	0,316	0,008	0,928	15,953
41	1	1,271	0,388	0,305	0,556	1,622	0,203	26,834
42	1	0	0,085	Infinity	0,078	0,421	0,517	3,535
43	1	0,437	0,183	0,418	0,262	0,725	0,394	11,269
44	1	0,735	0	0	0,155	8,602	0,003	6,213
45	1	1,14	0	0	0,364	11,765	0,001	9,64
46	1	0,277	0,317	1,146	0,31	0,003	0,953	15,496
47	1	0	0,207	Infinity	0,166	0,863	0,353	8,59
48	1	0,697	0,079	0,114	0,282	4,551	0,033	9,182
49	1	0,747	0,135	0,181	0,342	1,522	0,217	11,93
50	1	6,873	0,727	0,106	1,048	3,692	0,055	88,277
51	1	1,481	0,462	0,312	0,617	1,581	0,209	31,664
52	1	0,841	1,157	1,375	1,085	0,109	0,742	55,065
53	1	1,534	0,638	0,416	0,823	0,391	0,532	39,428
54	1	1,253	0,357	0,285	0,557	1,653	0,199	25,388
55	1	0	1,074	Infinity	0,761	2,029	0,154	44,513
56	1	0,225	0,947	4,2	0,8	0,673	0,412	41,147
57	1	0	0,322	Infinity	0,266	1,076	0,3	13,364
58	1	0,517	2,074	4,007	1,183	2,711	0,1	90,344
59	1	0	1,02	Infinity	0,67	3,111	0,078	42,289
60	1	0,674	0,809	1,2	0,786	0,018	0,894	39,264
61	1	1,893	0,968	0,512	1,07	0,305	0,581	56,157
62	1	1,239	1,092	0,881	1,114	0,011	0,917	55,748
63	1	1,526	1,07	0,701	1,224	0,172	0,678	57,284
64	1	2,674	0,525	0,196	0,707	1,614	0,204	44,356
65	1	2,464	0,34	0,138	0,86	4,523	0,033	34,929
66	1	1,452	0,66	0,455	0,967	1,073	0,3	39,653
67	1	1,069	0,865	0,81	0,907	0,042	0,838	44,918
68	1	0,849	0,987	1,162	0,945	0,018	0,892	48,092

69	1	0	1,481	Infinity	1,134	2,944	0,086	61,389
70	1	0	0,886	Infinity	0,785	1,096	0,295	36,733
71	1	30,586	0,719	0,023	1,008	2,844	0,092	288,477
72	1	1,381	0,23	0,167	0,598	3,568	0,059	21,232
73	1	1,274	1,38	1,083	1,346	0,007	0,935	67,978
74	1	0	0,418	Infinity	0,312	1,147	0,284	17,312
75	1	0	0,929	Infinity	0,696	1,116	0,291	38,531
76	1	3,267	0,809	0,248	1,102	0,966	0,326	61,188
77	1	3,21	0,799	0,249	1,082	1,35	0,245	60,277
78	1	2,395	0,354	0,148	0,864	2,974	0,085	34,926
79	1	4,629	0,506	0,109	1,303	6,737	0,009	60,145
80	1	6,981	0,34	0,049	0,523	2,992	0,084	73,13
81	1	5,595	0,877	0,157	1,627	2,198	0,138	83,687
82	1	1,511	1,538	1,018	1,529	0	0,99	76,525
83	1	3,125	1,157	0,37	1,364	0,4	0,527	74,395
84	1	2,39	1	0,418	1,26	0,656	0,418	61,662
85	1	0,967	5,016	5,186	4,745	0,179	0,672	216,142
86	1	0	5,776	Infinity	5,602	0,901	0,343	239,468
87	1	9,629	1,765	0,183	2,638	2,313	0,128	154,599
88	1	3,687	1,95	0,529	2,289	0,227	0,634	112,04
89	1	0	4,992	Infinity	3,356	0,425	0,515	206,939
90	1	34,352	6,137	0,179	7,082	0,371	0,542	544,939
91	1	0,939	5,287	5,628	3,754	0,702	0,402	227,137
92	1	0,422	5,606	13,291	4,72	2,217	0,137	235,966
93	1	1,57	1,281	0,816	1,338	0,042	0,838	66,384
94	1	0	2,26	Infinity	1,911	1,394	0,238	93,706
95	1	0	5,55	Infinity	3,362	2,366	0,124	230,078
96	1	0	7,875	Infinity	5,422	2,364	0,124	326,499
97	1	1,851	1,337	0,723	1,364	0,022	0,881	71,103
98	1	0,461	5,098	11,057	3,323	6,041	0,014	215,25
99	1	3,153	2,973	0,943	3,016	0,003	0,956	149,923
100	1	1,519	1,177	0,775	1,221	0,017	0,895	61,637
101	1	3,622	1,48	0,409	1,539	0,073	0,787	91,977
102	1	8,407	0,616	0,073	1,172	6,976	0,008	96,637
103	1	2,01	1,032	0,513	1,366	0,599	0,439	59,781
104	1	0,213	1,408	6,619	0,909	3,724	0,054	60,154
105	1	1,066	0,882	0,828	0,938	0,021	0,884	45,603
106	1	1,655	0,899	0,543	1,006	0,268	0,605	51,264
107	1	25,836	1,148	0,044	1,382	0,438	0,508	266,104

108	1	10,797	1,229	0,114	1,593	0,404	0,525	142,278
109	1	0	1,835	Infinity	1,117	2,839	0,092	76,066
110	1	3,597	1,084	0,301	1,763	0,84	0,359	75,348
111	1	3,412	0,395	0,116	1,029	3,855	0,05	45,253
112	1	0,668	1,143	1,711	1,087	0,055	0,814	53,048
113	1	1,391	1,528	1,099	1,486	0,012	0,912	75,132
114	1	4,871	1,196	0,245	1,98	3,254	0,071	90,768
115	1	0,838	1,797	2,144	1,576	0,372	0,542	81,569
116	1	2,032	5,516	2,715	4,12	0,836	0,361	245,856
117	1	1,526	1,12	0,734	1,232	0,074	0,785	59,322
118	1	28,032	3,661	0,131	5,293	1,705	0,192	388,854
119	1	1,328	2,557	1,925	2,075	0,062	0,803	117,237
120	1	12,862	2,646	0,206	4,083	0,652	0,419	218,46
121	1	0	4,844	Infinity	4,477	2,15	0,143	200,817
122	1	3,207	5,295	1,651	4,962	0,097	0,756	246,646
123	1	3,449	0,989	0,287	1,597	0,772	0,38	70,16
124	1	2,269	0,883	0,389	0,431	-1,584	1	55,797
125	1	0	6,327	Infinity	7,256	0,23	0,632	262,289
126	1	2,172	4,258	1,96	3,93	0,148	0,701	194,898
127	1	4,746	11,455	2,414	8,94	0,461	0,497	515,02
128	1	2,559	5,832	2,278	5,279	0,018	0,892	263,413
129	1	22,279	0,991	0,045	8,355	2,994	0,084	229,528
130	1	15,356	0,781	0,051	4,275	0,731	0,393	162,244
131	1	0	2,861	Infinity	3,32	1,182	0,277	118,594
132	1	21,496	2,19	0,102	3,812	0,811	0,368	272,612
133	1	0	4,618	Infinity	3,262	1,999	0,157	191,435
134	1	0	1,202	Infinity	0,99	1,37	0,242	49,814
135	1	6,147	3,293	0,536	3,622	0,079	0,778	188,512
136	1	0	7,884	Infinity	7,957	0,337	0,562	326,858
137	1	0	3,656	Infinity	1,696	3,557	0,059	151,582
138	1	10,214	1,899	0,186	2,636	0,707	0,401	165,13
139	1	6,139	13,5	2,199	11,918	0,237	0,626	611,592
140	1	0	5,707	Infinity	2,981	0,23	0,632	236,598
135	1	6,147	3,293	0,536	3,622	0,079	0,778	188,512
136	1	0	7,884	Infinity	7,957	0,337	0,562	326,858
137	1	0	3,656	Infinity	1,696	3,557	0,059	151,582
138	1	10,214	1,899	0,186	2,636	0,707	0,401	165,13
139	1	6,139	13,5	2,199	11,918	0,237	0,626	611,592
140	1	0	5,707	Infinity	2,981	0,23	0,632	236,598

141	1	0,561	2,832	5,046	1,326	0,495	0,482	122,172
142	1	0	4,745	Infinity	3,313	0,806	0,369	196,699
143	1	8,682	1,91	0,22	2,75	0,949	0,33	152,624
144	1	0	2,83	Infinity	2,092	1,417	0,234	117,308
145	1	4,484	16,11	3,593	14,076	0,054	0,815	705,791
146	1	22,175	1,163	0,052	1,488	1,689	0,194	235,743
147	1	0	1,944	Infinity	2,376	0,766	0,382	80,608
148	1	62,845	2,829	0,045	5,805	2,099	0,147	648,803
149	1	164,704	1,145	0,007	1,402	4,293	0,038	1440,452
150	1	0	2,054	Infinity	1,436	1,492	0,222	85,142
151	1	4,109	3,992	0,971	4,021	0,001	0,979	200,238
152	1	5,141	7,015	1,365	6,583	0,072	0,789	334,32
153	1	0	9,227	Infinity	7,963	5,114	0,024	382,527
154	1	8,225	1,162	0,141	1,369	0,873	0,35	117,746
**Site**	**Partition**	**alpha**	**beta**	**omega**	**alpha=beta**	**LRT**	**p-value**	**Total branch length**

Supplementary material S2. The results of full FEL analysis with negative (dark gray) and positive (green) selection sites identified in CD2v protein (*EP402R).*

Site - Site Position; **Partition** - Partition that site belong to; **alpha** - Synonymous substitution rate at a site; **beta** - Non-synonymous substitution rate at a site; **omega** - Ratio of nonsynonymous to synonymous substitution rate; **alpha=beta** - The rate estimate under the neutral model; **LRT** - Likelihood ration test statistic for beta = alpha, versus beta & neq, alpha; **p-value** - the p-value threshold to use when testing for selection; **Total branch length** - The total length of branches contributing to inference at this site, and used to scale dN-dS.

Site	Partition	alpha	beta	omega	alpha=beta	LRT	p-value	Total branch length
1	1	0	0,002	Infinity	0,002	0,011	0,918	0,243
2	1	0,05	0,256	5,147	0,137	2,116	0,146	34,567
3	1	0,133	0,099	0,743	0,111	0,092	0,762	17,306
4	1	0,634	0,359	0,566	0,425	0,556	0,456	68,098
5	1	0	0,15	Infinity	0,091	2,351	0,125	19,239
6	1	0,048	0,107	2,219	0,082	0,395	0,529	15,399
7	1	0,133	0,083	0,625	0,09	0,066	0,797	15,286
8	1	0,04	0,135	3,359	0,099	0,339	0,56	18,749
9	1	1,796	0,171	0,095	0,304	6,309	0,012	84,322
10	1	1,179	0,239	0,203	0,357	2,162	0,141	71,652
11	1	0,575	0,15	0,261	0,223	1,996	0,158	39,256
12	1	0	0,229	Infinity	0,2	0,542	0,461	29,417
13	1	0,357	0,102	0,287	0,164	1,979	0,159	25,541
14	1	0,125	0,046	0,369	0,068	0,881	0,348	10,239
15	1	0	0,095	Infinity	0,074	1,598	0,206	12,132
16	1	0,486	0,202	0,415	0,24	0,684	0,408	42,777
17	1	0,001	0,088	160,211	0,061	1,505	0,22	11,308
18	1	0,082	0,199	2,432	0,166	0,479	0,489	28,345
19	1	0,774	0,012	0,016	0,119	14,447	0	28,481
20	1	0,931	0,133	0,143	0,245	4,892	0,027	49,459
21	1	0,482	0,144	0,299	0,206	1,151	0,283	35,277
22	1	11,369	0,253	0,022	0,361	4,851	0,028	427,53
23	1	0,172	0,109	0,632	0,138	0,123	0,726	19,94
24	1	0,751	0,24	0,319	0,334	1,082	0,298	56,882
25	1	5,261	0,31	0,059	0,47	4,209	0,04	222,573
26	1	0,173	0,035	0,204	0,084	1,612	0,204	10,558
27	1	0,149	0,195	1,308	0,175	0,048	0,827	30,203
28	1	0,089	0,221	2,471	0,182	0,58	0,446	31,47
29	1	0	0,141	Infinity	0,105	0,561	0,454	18,123
30	1	0,003	0,218	71,627	0,167	0,478	0,489	28,113

31	1	0,2	0,16	0,798	0,169	0,028	0,867	27,452
32	1	0,99	0,091	0,092	0,268	7,679	0,006	46,053
33	1	0,24	0,134	0,558	0,173	0,474	0,491	25,474
34	1	0,064	0,361	5,61	0,215	1,973	0,16	48,567
35	1	0,838	0,11	0,131	0,247	2,582	0,108	43,235
36	1	0,354	0,18	0,509	0,223	0,39	0,532	35,448
37	1	0,477	0,373	0,782	0,385	0,04	0,842	64,45
38	1	0,128	0,237	1,843	0,222	0,104	0,747	34,824
39	1	0	0,231	Infinity	0,173	2,852	0,091	29,665
40	1	0,229	0,316	1,384	0,295	0,056	0,813	48,539
41	1	0,476	0,525	1,102	0,504	0,011	0,917	83,923
42	1	0,75	0,237	0,316	0,351	2,823	0,093	56,434
43	1	0,331	0,195	0,588	0,261	0,246	0,62	36,49
44	1	3,636	0,349	0,096	0,438	1,821	0,177	171,186
45	1	0,391	0,271	0,693	0,284	0,024	0,876	48,298
46	1	0,615	0,235	0,382	0,271	0,535	0,464	51,443
47	1	0,802	0,466	0,582	0,533	0,241	0,623	87,691
48	1	0,273	0,526	1,929	0,454	0,217	0,641	76,93
49	1	0,311	0,305	0,982	0,307	0	0,986	49,932
50	1	0,759	0,048	0,064	0,123	4,603	0,032	32,557
51	1	0	0,114	Infinity	0,092	1,272	0,259	14,606
52	1	0,324	0,163	0,502	0,197	0,305	0,581	32,159
53	1	0,373	0,271	0,726	0,307	0,081	0,776	47,732
54	1	2,135	0,207	0,097	0,295	2,84	0,092	100,814
55	1	0,222	0,297	1,334	0,289	0,018	0,893	45,763
56	1	0,714	0,16	0,224	0,265	2,174	0,14	45,34
57	1	0,07	0,243	3,458	0,158	0,647	0,421	33,649
58	1	0,123	0,092	0,749	0,099	0,05	0,823	16,032
59	1	7,169	0,354	0,049	0,375	3,289	0,07	294,512
60	1	0,308	0,111	0,36	0,167	1,045	0,307	24,927
61	1	0,294	0,148	0,503	0,184	0,388	0,533	29,208
62	1	0,438	0,223	0,509	0,269	0,389	0,533	43,762
63	1	0,263	0,121	0,461	0,147	0,291	0,589	24,673
64	1	0	0,998	Infinity	0,459	2,492	0,114	127,993
65	1	0,652	0,294	0,451	0,333	0,381	0,537	60,335
66	1	0,367	0,308	0,84	0,322	0,025	0,873	52,323
67	1	1,398	0,295	0,211	0,553	4,853	0,028	86,494
68	1	0,373	0,183	0,49	0,235	0,515	0,473	36,401
69	1	1,588	0,245	0,155	0,367	3,562	0,059	86,655

70	1	0,363	0,308	0,849	0,325	0,03	0,863	52,091
71	1	0,778	0,138	0,177	0,211	1,622	0,203	44,711
72	1	0,673	0,263	0,39	0,252	0,13	0,718	57,063
73	1	0,562	0,349	0,621	0,386	0,181	0,67	64,271
74	1	0,43	0,371	0,863	0,392	0,017	0,897	62,586
75	1	0,477	0,318	0,665	0,367	0,148	0,7	57,339
76	1	1,239	0,088	0,071	0,251	7,981	0,005	54,322
77	1	0,336	0,282	0,841	0,288	0,01	0,92	47,888
78	1	0,038	0,45	11,894	0,339	1,515	0,218	59,052
79	1	0,319	0,504	1,58	0,458	0,137	0,711	75,686
80	1	0,279	0,168	0,601	0,191	0,155	0,694	31,187
61	1	0,294	0,148	0,503	0,184	0,388	0,533	29,208
62	1	0,438	0,223	0,509	0,269	0,389	0,533	43,762
63	1	0,263	0,121	0,461	0,147	0,291	0,589	24,673
64	1	0	0,998	Infinity	0,459	2,492	0,114	127,993
65	1	0,652	0,294	0,451	0,333	0,381	0,537	60,335
66	1	0,367	0,308	0,84	0,322	0,025	0,873	52,323
67	1	1,398	0,295	0,211	0,553	4,853	0,028	86,494
68	1	0,373	0,183	0,49	0,235	0,515	0,473	36,401
69	1	1,588	0,245	0,155	0,367	3,562	0,059	86,655
70	1	0,363	0,308	0,849	0,325	0,03	0,863	52,091
71	1	0,778	0,138	0,177	0,211	1,622	0,203	44,711
72	1	0,673	0,263	0,39	0,252	0,13	0,718	57,063
73	1	0,562	0,349	0,621	0,386	0,181	0,67	64,271
74	1	0,43	0,371	0,863	0,392	0,017	0,897	62,586
75	1	0,477	0,318	0,665	0,367	0,148	0,7	57,339
76	1	1,239	0,088	0,071	0,251	7,981	0,005	54,322
77	1	0,336	0,282	0,841	0,288	0,01	0,92	47,888
78	1	0,038	0,45	11,894	0,339	1,515	0,218	59,052
79	1	0,319	0,504	1,58	0,458	0,137	0,711	75,686
80	1	0,279	0,168	0,601	0,191	0,155	0,694	31,187
81	1	2,941	0,161	0,055	0,685	6,658	0,01	122,85
82	1	0,92	0,227	0,247	0,253	0,973	0,324	61,122
83	1	0,202	0,152	0,75	0,167	0,046	0,829	26,509
84	1	0,851	0,107	0,125	0,132	1,997	0,158	43,255
85	1	0,649	0,279	0,43	0,39	0,985	0,321	58,347
86	1	0,197	0,217	1,105	0,213	0,008	0,929	34,706
87	1	0,663	0,214	0,323	0,298	1,214	0,271	50,5
88	1	0	0,303	Infinity	0,203	2,336	0,126	38,828

89	1	0,486	0,363	0,746	0,395	0,048	0,826	63,431
90	1	0,371	0,249	0,672	0,295	0,125	0,724	44,903
91	1	0,861	0,144	0,168	0,28	3,44	0,064	48,454
92	1	0,311	0,31	0,997	0,31	0	0,998	50,568
93	1	0,067	0,215	3,204	0,15	0,725	0,394	29,85
94	1	0,656	0,162	0,247	0,231	0,994	0,319	43,624
95	1	0,427	0,552	1,293	0,522	0,031	0,861	85,717
96	1	0,207	0,274	1,323	0,25	0,04	0,841	42,277
97	1	0,056	0,408	7,317	0,278	0,471	0,493	54,276
98	1	0,685	0,097	0,142	0,229	2,421	0,12	36,235
99	1	0,761	0,258	0,339	0,403	1,111	0,292	59,543
100	1	0,289	0,208	0,719	0,228	0,071	0,789	36,654
101	1	0,352	0,076	0,216	0,125	1,268	0,26	22,012
102	1	0,083	0,273	3,303	0,242	0,146	0,702	37,865
103	1	0,357	0,216	0,605	0,228	0,097	0,755	40,102
104	1	0,77	0,249	0,323	0,341	1,193	0,275	58,706
105	1	2,874	0,164	0,057	0,191	0,548	0,459	120,856
106	1	0,306	0,182	0,596	0,213	0,154	0,695	34,048
107	1	0,69	0,183	0,265	0,306	2,113	0,146	47,397
108	1	1,016	0,186	0,183	0,349	2,81	0,094	59,141
109	1	14,913	0,2	0,013	0,387	8,044	0,005	543,845
110	1	0,121	0,293	2,429	0,24	0,572	0,45	41,827
111	1	0,242	0,239	0,99	0,241	0	0,993	39,084
112	1	0,902	0,317	0,352	0,396	1,143	0,285	72,066
113	1	0,23	0,119	0,516	0,146	0,246	0,62	23,253
114	1	0,833	0,294	0,353	0,444	0,715	0,398	66,691
115	1	0,435	0,398	0,915	0,41	0,005	0,944	66,159
116	1	1,219	0,204	0,168	0,546	3,667	0,055	68,591
117	1	0,493	0,217	0,441	0,298	0,737	0,391	45,011
118	1	2,391	0,094	0,039	0,195	5,465	0,019	95,145
119	1	0,686	0,256	0,374	0,358	1,137	0,286	56,737
120	1	0,539	0,183	0,339	0,258	0,901	0,342	42,181
121	1	0,249	0,327	1,313	0,302	0,066	0,797	50,597
122	1	0,384	0,259	0,674	0,31	0,159	0,69	46,5
123	1	0,675	0,246	0,364	0,361	1,326	0,249	54,98
124	1	1,38	0,237	0,172	0,423	2,302	0,129	78,329
125	1	0,617	0,354	0,573	0,416	0,259	0,611	66,884
126	1	0,712	0,21	0,295	0,273	0,877	0,349	51,734
127	1	0,036	0,407	11,352	0,322	1,114	0,291	53,455

128	1	0,338	0,393	1,164	0,374	0,016	0,9	62,125
129	1	0,384	0,338	0,881	0,351	0,014	0,905	56,724
130	1	0,704	0,231	0,328	0,323	0,868	0,351	54,069
131	1	0,227	0,405	1,789	0,333	0,302	0,583	59,841
132	1	0,565	0,133	0,236	0,225	1,954	0,162	36,712
133	1	0,193	0,127	0,659	0,142	0,071	0,79	22,982
134	1	0	0,3	Infinity	0,161	3,531	0,06	38,526
135	1	0,387	0,141	0,363	0,191	0,531	0,466	31,477
136	1	2,903	0,145	0,05	0,366	4,191	0,041	119,444
137	1	0,891	0,15	0,169	0,262	2,215	0,137	50,245
138	1	0,481	0,233	0,484	0,33	0,736	0,391	46,56
139	1	0,699	0,332	0,476	0,367	0,261	0,61	66,911
140	1	1,585	0,166	0,105	0,369	5,526	0,019	76,397
141	1	0,598	0,211	0,353	0,316	0,799	0,371	47,902
142	1	1,14	0,097	0,085	0,208	5,06	0,024	52,105
143	1	0,117	0,377	3,211	0,248	0,498	0,48	52,462
144	1	0,919	0,204	0,222	0,313	1,51	0,219	58,109
145	1	0,786	0,293	0,373	0,391	0,99	0,32	64,902
146	1	0,481	0,343	0,712	0,375	0,083	0,773	60,679
147	1	0,096	0,29	3,028	0,219	0,598	0,439	40,522
148	1	1,458	0,591	0,406	0,747	0,817	0,366	126,531
149	1	1,69	0,376	0,222	0,549	1,844	0,174	106,917
150	1	0,482	0,222	0,461	0,278	0,626	0,429	45,265
151	1	0,133	0,326	2,448	0,262	0,446	0,504	46,427
152	1	0,603	0,633	1,049	0,624	0,003	0,957	102,168
153	1	3,2	0,689	0,215	1,024	1,355	0,244	199,614
154	1	0,173	0,606	3,503	0,452	0,875	0,349	83,806
155	1	0,648	0,346	0,534	0,412	0,38	0,537	66,874
156	1	1,111	0,776	0,698	0,867	0,112	0,737	138,163
157	1	0,563	0,634	1,126	0,61	0,012	0,912	100,887
158	1	0,947	0,522	0,551	0,605	0,408	0,523	99,918
159	1	0,423	0,315	0,745	0,339	0,077	0,781	55,129
160	1	2,875	0,45	0,157	0,755	2,138	0,144	157,649
161	1	1,396	0,497	0,356	0,578	0,573	0,449	112,285
162	1	0,509	0,528	1,039	0,521	0,001	0,972	85,422
163	1	0,294	0,285	0,968	0,287	0	0,983	46,772
164	1	2,761	0,352	0,127	0,688	3,363	0,067	141,038
165	1	0,528	0,216	0,409	0,296	0,501	0,479	46,087
166	1	0,76	0,305	0,401	0,407	0,703	0,402	65,537

167	1	6,934	0,381	0,055	1,331	2,885	0,089	289,796
168	1	0,507	0,489	0,964	0,495	0,001	0,97	80,393
169	1	1,046	0,574	0,549	0,654	0,297	0,586	110,028
170	1	2,271	0,417	0,184	0,528	1,29	0,256	132,402
171	1	0,232	0,459	1,977	0,386	0,361	0,548	66,995
172	1	0,636	0,199	0,312	0,302	0,742	0,389	47,613
173	1	0,105	1,16	11,096	0,706	2,102	0,147	152,401
174	1	2,549	0,36	0,141	0,742	3,878	0,049	134,739
175	1	0,262	0,563	2,149	0,43	0,427	0,513	81,394
176	1	1,626	0,739	0,455	1,007	0,93	0,335	151,36
177	1	0,109	0,679	6,207	0,402	1,414	0,234	90,851
178	1	0,308	0,883	2,87	0,679	0,87	0,351	123,948
179	1	0,559	0,231	0,414	0,321	0,635	0,425	49,088
180	1	0,745	0,331	0,444	0,416	0,707	0,401	68,297
181	1	0,214	0,163	0,759	0,179	0,048	0,827	28,337
182	1	2,234	0,427	0,191	0,669	1,695	0,193	132,397
183	1	0,475	0,331	0,696	0,346	0,031	0,861	59,007
184	1	0,462	0,393	0,852	0,419	0,013	0,909	66,491
185	1	0,374	0,18	0,48	0,234	0,24	0,624	36,069
186	1	1,91	0,606	0,317	0,821	0,452	0,501	144,134
187	1	0,395	0,486	1,23	0,439	0,041	0,84	76,085
188	1	1,068	0,697	0,653	0,783	0,135	0,713	126,561
189	1	2,348	0,29	0,123	0,578	3,352	0,067	118,785
190	1	0,587	0,443	0,754	0,512	0,098	0,755	77,2
191	1	1,169	0,283	0,242	0,507	2,233	0,135	76,943
192	1	3,555	0,426	0,12	0,904	2,342	0,126	178,194
193	1	0,974	0,552	0,566	0,671	0,391	0,532	104,657
194	1	0,214	0,64	2,989	0,444	0,712	0,399	89,554
195	1	4,06	0,458	0,113	0,593	2,442	0,118	199,828
196	1	1,253	0,474	0,378	0,696	0,915	0,339	104,375
197	1	0,451	0,229	0,507	0,31	0,283	0,595	45,001
198	1	2,684	0,535	0,199	0,998	2,167	0,141	161,904
199	1	0	0,7	Infinity	0,405	1,859	0,173	89,796
200	1	2,675	0,367	0,137	0,785	3,308	0,069	140,074
201	1	0,558	0,39	0,7	0,436	0,091	0,762	69,469
202	1	0,254	1,629	6,407	1,051	1,324	0,25	217,787
203	1	1,038	1,163	1,12	1,112	0,018	0,894	185,212
204	1	0,847	0,465	0,548	0,582	0,378	0,539	89,035
205	1	1,508	0,444	0,295	0,571	0,915	0,339	109,41

206	1	0,794	0,176	0,222	0,335	0,866	0,352	50,212
207	1	0,448	0,829	1,851	0,701	0,336	0,562	121,891
208	1	1,204	0,464	0,386	0,782	1,107	0,293	101,404
209	1	0,667	0,532	0,797	0,573	0,035	0,852	91,354
210	1	3,217	0,931	0,289	1,243	1,191	0,275	231,166
211	1	1,543	0,654	0,424	0,781	0,341	0,559	137,454
212	1	1,198	0,693	0,579	0,824	0,366	0,545	130,573
213	1	1,871	0,417	0,223	0,881	2,442	0,118	118,566
214	1	0,896	0,452	0,504	0,548	0,373	0,541	89,07
215	1	1,204	0,595	0,494	0,829	0,497	0,481	118,143
216	1	0,759	0,608	0,801	0,651	0,031	0,859	104,327
217	1	7,132	0,654	0,092	1,359	4,35	0,037	331,72
218	1	1,953	0,395	0,202	0,993	1,895	0,169	118,531
219	1	0,443	0,485	1,095	0,471	0,007	0,933	77,634
220	1	0,291	1,068	3,671	0,682	2,258	0,133	147,058
221	1	1,266	0,543	0,429	0,696	0,73	0,393	113,675
222	1	1,492	0,634	0,425	0,855	1,033	0,309	133,13
223	1	0,267	0,57	2,134	0,461	0,219	0,64	82,351
224	1	0,743	0,247	0,332	0,376	1,54	0,215	57,47
225	1	0,951	0,267	0,281	0,435	1,38	0,24	67,368
226	1	0,313	0,517	1,651	0,447	0,15	0,698	77,236
227	1	1,306	1,308	1,002	1,309	0	1	213,231
228	1	1,315	1,051	0,799	1,125	0,04	0,841	180,539
229	1	2,293	0,391	0,171	0,944	4,082	0,043	129,835
230	1	0,878	0,499	0,568	0,681	0,404	0,525	94,567
231	1	1,039	0,458	0,441	0,722	0,77	0,38	94,872
232	1	1,878	0,867	0,461	1,165	0,531	0,466	176,424
233	1	1,132	0,176	0,156	0,512	1,943	0,163	61,95
234	1	0,815	0,523	0,641	0,664	0,149	0,699	95,379
235	1	1,007	0,793	0,787	0,881	0,071	0,79	136,692
236	1	0,388	0,248	0,639	0,29	0,066	0,797	45,317
237	1	1,105	0,792	0,716	0,893	0,063	0,802	139,933
238	1	1,768	0,879	0,498	1,081	0,203	0,652	174,249
239	1	0,298	0,206	0,692	0,231	0,076	0,783	36,759
240	1	0,433	0,711	1,643	0,63	0,144	0,705	106,282
241	1	0,445	1,023	2,298	0,743	0,483	0,487	146,769
242	1	0,955	1,613	1,688	1,404	0,161	0,688	240,157
243	1	0,585	0,394	0,673	0,437	0,096	0,756	70,863
244	1	0,78	0,645	0,827	0,675	0,041	0,84	109,83

245	1	9,576	0,222	0,023	0,807	6,945	0,008	361,255
246	1	2,103	1,279	0,608	1,491	0,254	0,614	237,225
247	1	1,527	0,432	0,283	0,642	1,638	0,201	108,487
248	1	0,587	0,17	0,29	0,231	0,658	0,417	42,222
249	1	0,298	0,784	2,63	0,584	0,319	0,572	110,969
250	1	10,35	1,74	0,168	2,661	0,581	0,446	582,885
251	1	16,493	1,47	0,089	1,912	2,881	0,09	761,722
252	1	0	1,924	Infinity	1,104	5,665	0,017	246,805
253	1	0,252	1,101	4,363	0,808	1,227	0,268	149,977
254	1	3,117	0,178	0,057	0,761	5,95	0,015	131,149
255	1	0,802	0,852	1,062	0,845	0,002	0,965	137,176
256	1	2,293	1,731	0,755	1,831	0,068	0,794	301,766
257	1	0,411	2,361	5,742	2,104	1,301	0,254	317,221
258	1	1,486	6,542	4,402	5,753	0,778	0,378	890,846
259	1	0,51	3,182	6,242	1,827	2,47	0,116	425,961
260	1	3,901	0,164	0,042	0,349	2,553	0,11	156,615
261	1	0,84	0,944	1,124	0,901	0,024	0,876	150,317
262	1	0,468	2,326	4,969	1,243	1,515	0,218	314,714
263	1	0,268	1,98	7,388	1,483	2,664	0,103	263,292
264	1	4,013	1,236	0,308	1,615	0,607	0,436	298,031
265	1	0,973	0,587	0,603	0,71	0,314	0,575	109,111
266	1	6,817	2,418	0,355	3,249	0,353	0,552	547,014
267	1	0,427	3,923	9,194	3,846	2,552	0,11	518,101
268	1	0,805	2,206	2,741	1,656	1,026	0,311	310,915
269	1	2,816	3,773	1,34	3,469	0,06	0,807	581,93
270	1	5,177	1,176	0,227	2,183	2,416	0,12	330,827
271	1	1,627	1,136	0,699	1,318	0,097	0,756	202,313
272	1	0,809	2,751	3,402	1,864	0,495	0,482	380,995
273	1	2,623	0,728	0,277	0,966	1,067	0,302	184,507
274	1	1,179	3,422	2,903	2,522	1,415	0,234	479,948
275	1	1,269	1,101	0,868	1,183	0,024	0,876	185,292
276	1	4,701	0,489	0,104	1,708	2,62	0,106	226,079
277	1	1,268	3,848	3,034	2,71	0,388	0,533	537,71
278	1	0,99	1,693	1,71	1,467	0,202	0,653	251,532
279	1	20,555	3,384	0,165	3,871	1,278	0,258	1148,359
280	1	1,425	1,648	1,156	1,576	0,022	0,881	260,905
281	1	1,717	3,657	2,13	2,867	0,455	0,5	528,77
282	1	0,683	4,379	6,411	2,672	1,841	0,175	585,517
283	1	1,123	1,068	0,951	1,091	0,004	0,951	176,013

284	1	1,73	1,749	1,011	1,745	0	0,993	284,466
285	1	0,66	3,518	5,332	3,337	0,453	0,501	474,212
286	1	1,613	2,718	1,685	1,892	0,174	0,677	404,702
287	1	0,876	1,075	1,226	0,975	0,057	0,811	168,326
288	1	0,765	4,479	5,853	1,553	1,918	0,166	601,25
289	1	1,134	0,963	0,849	1,019	0,025	0,875	162,875
290	1	1,533	0,914	0,596	1,085	0,376	0,54	170,504
291	1	1,139	3,709	3,257	2,488	1,44	0,23	515,378
292	1	0,492	2,858	5,806	1,543	3,245	0,072	383,715
293	1	1,256	2,839	2,26	1,692	0,291	0,59	407,865
294	1	1,679	2,067	1,231	1,93	0,045	0,832	323,472
295	1	0,775	0,903	1,165	0,815	0,029	0,864	142,801
296	1	0,679	4,604	6,779	3,994	2,4	0,121	614,29
297	1	0,782	1,922	2,458	1,514	0,866	0,352	273,765
298	1	6,037	0,814	0,135	1,567	2,544	0,111	314,186
299	1	10,177	1,472	0,145	1,739	1,63	0,202	542,497
300	1	1,116	2,04	1,828	1,724	0,139	0,709	300,444
301	1	0,658	1,125	1,709	1,001	0,335	0,563	167,15
302	1	0,589	3,221	5,468	1,424	2,893	0,089	433,699
303	1	1,152	0,806	0,7	0,978	0,127	0,722	143,498
304	1	8,491	0,811	0,096	3,773	2,881	0,09	399,155
305	1	2,054	1,35	0,657	1,623	0,085	0,77	244,585
306	1	4,766	1,936	0,406	2,442	1,055	0,304	413,979
307	1	30,142	0,37	0,012	1,543	9,385	0,002	1094,872
308	1	36,476	0,981	0,027	3,112	4,534	0,033	1393,434
309	1	15,594	0,278	0,018	1,826	9,828	0,002	577,575
310	1	19,094	0,245	0,013	0,843	16,004	0	694,947
311	1	0,595	0,507	0,852	0,552	0,034	0,853	85,677
312	1	1,549	1,002	0,647	1,167	0,279	0,597	182,354
313	1	1,863	0,469	0,252	0,696	1,421	0,233	124,967
314	1	3,051	0,503	0,165	1,319	2,762	0,097	170,536
315	1	1,54	0,273	0,177	0,578	3,209	0,073	88,52
316	1	14,31	0,355	0,025	0,681	7,209	0,007	542,786
317	1	1,078	0,481	0,446	0,714	0,534	0,465	99,17
318	1	9,245	1,423	0,154	1,952	3,321	0,068	503,862
319	1	38,883	0,312	0,008	1,289	14,566	0	1391,161
320	1	32,872	1,578	0,048	2,81	3,135	0,077	1344,686
321	1	2,34	0,187	0,08	0,643	4,517	0,034	105,3
322	1	1,306	0,625	0,478	0,885	0,756	0,385	125,561

323	1	0,699	0,235	0,336	0,414	1,484	0,223	54,427
324	1	5,644	1,641	0,291	1,951	1,219	0,27	406,656
325	1	19,13	0,181	0,009	0,38	5,267	0,022	687,945
326	1	13,747	1,246	0,091	2,923	2,37	0,124	637,61
327	1	6,229	0,112	0,018	1,098	17,791	0	230,825
328	1	5,282	0,518	0,098	1,146	4,637	0,031	249,997
329	1	0,6	0,509	0,847	0,55	0,042	0,839	86,106
330	1	2,853	1,411	0,495	1,653	0,749	0,387	280,129
331	1	1,18	0,156	0,132	0,387	3,831	0,05	61,032
332	1	1,246	1,063	0,853	1,167	0,016	0,9	179,691
333	1	3,876	0,094	0,024	0,774	11,206	0,001	146,727
334	1	5,481	0	0	0,552	12,152	0	190,481
335	1	0,669	0,501	0,749	0,563	0,108	0,742	87,5
336	1	9,146	1,357	0,148	2,015	4,122	0,042	491,898
337	1	8,242	0,956	0,116	1,335	2,331	0,127	409,111
338	1	9,934	0,464	0,047	0,555	-1,428	1	404,737
339	1	1,006	0,368	0,366	0,624	1,43	0,232	82,192
340	1	4,998	0,984	0,197	1,608	3,243	0,072	299,894
341	1	0,936	0,549	0,587	0,688	0,34	0,56	103,028
342	1	1,065	2,475	2,325	2,182	0,378	0,539	354,528
343	1	10,014	0,377	0,038	1,001	0,686	0,407	396,357
344	1	1,055	0,588	0,557	0,782	0,438	0,508	112,079
345	1	5,73	0,334	0,058	1,116	5,773	0,016	242
346	1	0	1,004	Infinity	0,579	1,376	0,241	128,812
347	1	16,375	0,49	0,03	1,194	7,188	0,007	631,854
348	1	6,541	0,87	0,133	1,44	3,654	0,056	338,99
349	1	6,188	1,695	0,274	2,001	-4,99	1	432,532
350	1	3,273	0,886	0,271	1,349	0,999	0,318	227,413
351	1	3,45	1,79	0,519	2,083	0,187	0,665	349,52
352	1	0	0,862	Infinity	0,489	2,254	0,133	110,589
353	1	1,143	0,542	0,475	0,807	0,881	0,348	109,259
354	1	4,126	0,84	0,204	1,402	3,437	0,064	251,142
355	1	8,037	2,505	0,312	4,232	0,89	0,346	600,613
356	1	0,906	3,682	4,064	2,809	0,935	0,333	503,904
357	1	16,658	0,746	0,045	6,325	2,11	0,146	674,493
358	1	6,942	1,029	0,148	1,495	0,886	0,347	373,291
359	1	0	2,683	Infinity	1,634	4,097	0,043	344,13
360	1	4,566	0,974	0,213	1,753	1,899	0,168	283,626
361	1	36,552	6,442	0,176	10,691	1,759	0,185	2096,575

362	1	7,084	6,632	0,936	6,782	0,004	0,95	1096,927
363	1	8,155	4,556	0,559	5,706	0,209	0,648	867,869
364	1	9,071	1,387	0,153	2,452	2,759	0,097	493,181
365	1	6,045	3,282	0,543	4,016	0,208	0,648	631,169
366	1	7,462	2,905	0,389	3,746	0,573	0,449	632,047
367	1	2,857	1,136	0,398	1,53	0,7	0,403	245,072
368	1	2,675	3,843	1,437	3,503	0,036	0,85	586,004
369	1	9,184	7,376	0,803	7,972	0,03	0,862	1265,367
370	1	15,743	3,379	0,215	4,557	0,563	0,453	980,621
371	1	12,024	4,07	0,338	5,28	0,583	0,445	939,996
372	1	8,808	2,722	0,309	3,855	0,7	0,403	655,315
373	1	6,905	2,418	0,35	3,733	0,781	0,377	550,113
374	1	0	4,386	Infinity	2,973	2,117	0,146	562,673
375	1	1,701	1,934	1,137	1,77	0,002	0,969	307,272
376	1	26,857	6,826	0,254	11,28	0,869	0,351	1808,953
377	1	4,869	8,747	1,797	7,634	0,084	0,772	1291,299
378	1	4,414	7,317	1,658	6,754	0,073	0,786	1092,074
379	1	0	6,046	Infinity	5,233	1,505	0,22	775,64
380	1	8,648	0,914	0,106	1,486	1,079	0,299	417,836
381	1	3,581	4,044	1,129	3,909	0,003	0,958	643,165
382	1	6,342	2,097	0,331	2,36	0,218	0,64	489,442
383	1	0	4,307	Infinity	4,338	0,579	0,447	552,532
384	1	1,273	7,455	5,855	6,565	-0,023	1	1000,567
385	1	8,101	1,752	0,216	2,579	0,717	0,397	506,229
386	1	1,025	3,815	3,722	2,815	0,336	0,562	525,004
387	1	3,744	0,32	0,086	0	-1,05	1	171,188
388	1	6,981	1,437	0,206	1,891	0,335	0,563	426,953
389	1	5,308	0,379	0,071	0,812	0,232	0,63	233,075
390	1	2,541	0	0	2,104	0,065	0,799	88,317
391	1	3,821	0	0	2,203	0,06	0,807	132,785
392	1	0,249	0,368	1,478	0,292	0,039	0,844	55,81
393	1	4,415	1,212	0,275	2,162	0,129	0,72	308,942
394	1	0	0	NaN	0	0	1	0
395	1	4,738	0	0	2,004	0,033	0,857	164,646
396	1	0,467	0,833	1,784	0,741	0,121	0,728	123,056
397	1	0	0	NaN	0	0	1	0
398	1	1,458	0	0	0,518	1,244	0,265	50,678
399	1	0,093	0	0	0,035	0,03	0,863	3,234
400	1	0	0	NaN	0	0	1	0

Site	Partition	alpha	beta	omega	alpha=beta	LRT	p-value	Total branch length
401	1	0	0	NaN	0	0	1	0
402	1	0,902	0,624	0,692	0,675	0,027	0,87	111,418
403	1	0,39	0,208	0,532	0,247	0,053	0,818	40,194

Supplementary material S3. The protein alignments of ASFV C-type lectin (*EP153R*). The amino acids under the selective pressure according to the FEL analysis are marked by asterisk (A).

A

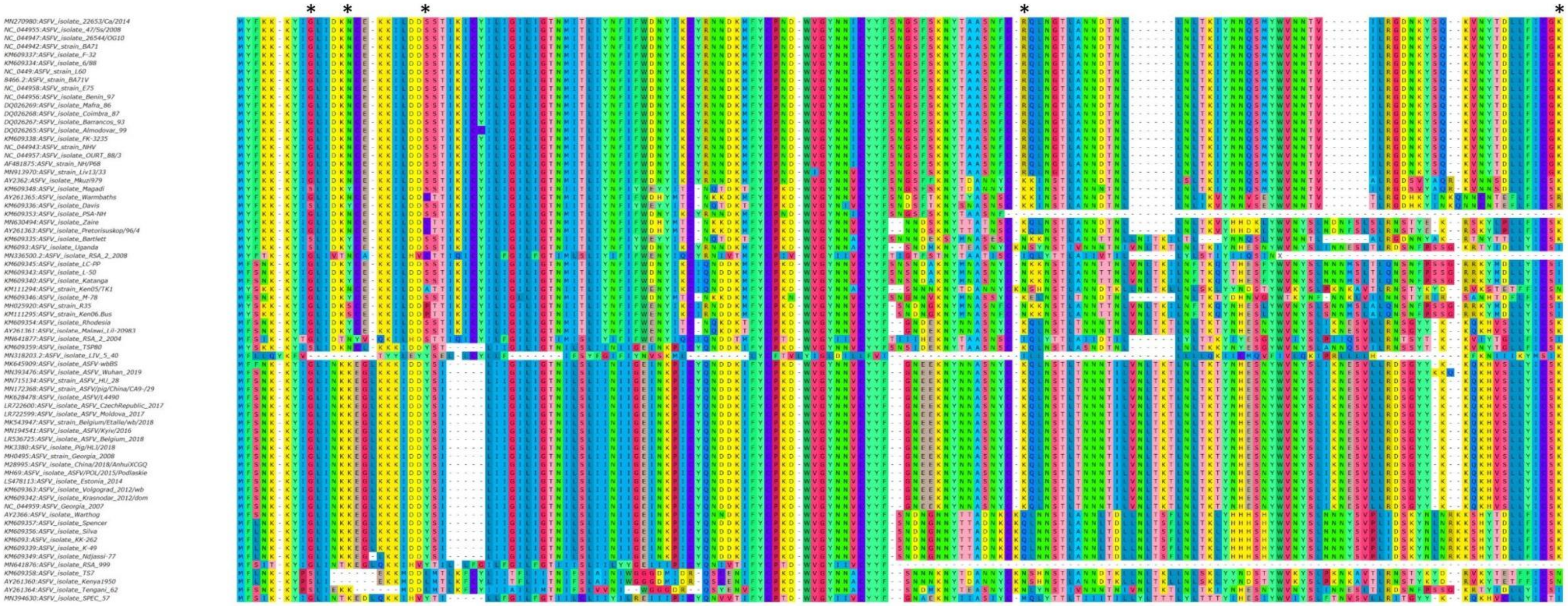

The protein alignments of ASFV CD2v (*EP402R*). The amino acids under the selective pressure according to the FEL analysis are marked by asterisk (B). The alignments were produced by UGENE v35.1 [52].

B

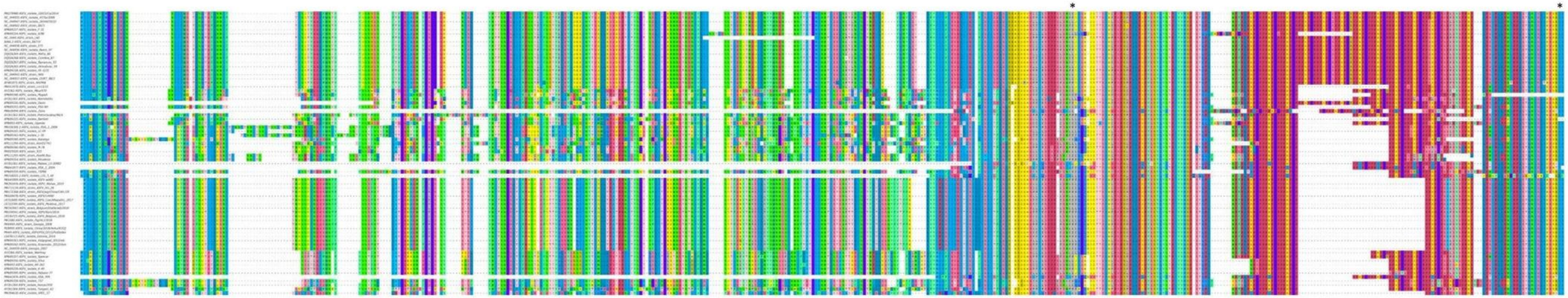

Supplementary material S4. The dataset of the ASFV *EP153R* (C-type lectin) and *EP402R* (CD2v) genes used in this study with the GenBank accession numbers and assigned genotypes.

ASFV strains/isolates	GenBank number *EP153R* (C-type lectin)/*EP402R* (CD2v)	p72 Genotype
22653/Ca/2014	MN270980.1	I
47/Ss/2008	NC_044955.1	I
26544/OG10_from_Italy	NC_044947.1	I
BA71	NC_044942.1	I
F-32	KM609337.1	I
691/88	KM609334.1	I
L60	NC_044941.1	I
BA71V	U18466.2	I
E75	NC_044958.1	I
Benin 97/1	NC_044956.1	I
Mafra_86	DQ026269.1	I
Coimbra_87	DQ026268.1	I
Barrancos_93	DQ026267.1	I
Almodovar_99	DQ026265.1	I
FK-32/135	KM609338.1	I
NHV	NC_044943.1	I
OURT_88/3	NC_044957.1	I
NH/P68	AF481875.1	I
Liv13/33	MN913970.1	I
Mkuzi 1979	AY261362.1	I
Magadi	KM609348.1	X
Warmbaths	AY261365.1	III
Davis	KM609336.1	X
PSA-1-NH	KM609353.1	I
Zaire	MN630494.1	XX
Pretorisuskop/96/4	AY261363.1	XX
Bartlett	KM609335.1	X
Uganda	KM609361.1	X
RSA_2_2008	MN336500.2	XXII
LC-PP	KM609345.1	I
L-50	KM609343.1	I
Katanga	KM609340.1	I
Ken05/Tk1	KM111294.1	X

M-78	KM609346.1	V
R35	MH025920.1	IX
Ken06.Bus	KM111295.1	IX
Rhodesia	KM609354.1	VIII
Malawi Lil-20/1	AY261361.1	VIII
RSA_2_2004	MN641877.1	XX
TSP-80	KM609359.1	X
LIV_5_40	MN318203.2	I
ASFV-wbBS01	MK645909.1	II
ASFV_Wuhan_2019-1	MN393476.1	II
HU_2018	MN715134.1	II
ASFV/pig/China/CAS19-01/2019	MN172368.1	II
ASFV/LT14/1490	MK628478.1	II
ASFV_Czech Republic_2017/1	LR722600.1	II
ASFV_Moldova_2017/1	LR722599.1	II
Belgium/Etalle/wb/2018	MK543947.1	II
ASFV/Kyiv/2016/131	MN194591.1	II
ASFV_Belgium_2018/1	LR536725.1	II
Pig/HLJ/2018	MK333180.1	II
Georgia_2008/1	MH910495.1	II
China/2018/AnhuiXCGQ	MK128995.1	II
ASFV/POL/2015/Podlaskie	MH681419.1	II
Estonia_2014	LS478113.1	II
Volgograd_2012/wb	KM609363.1	II
Krasnodar_2012/dom	KM609342.1	II
Georgia_2007/1	NC_044959.1	II
Warthog	AY261366.1	IV
Spenser	KM609357.1	untyped
Sylva-1	KM609356.1	I
KK-262	KM609341.1	I
K-49	KM609339.1	I
Ndjassi-77	KM609349.1	I
RSA_W1_1999	MN641876.1	IV
TS-7	KM609358.1	X
Kenya 1950	AY261360.1	X
Tengani 62	AY261364.1	V
SPEC_57	MN394630.2	III
