# Supplementary material for: Recombination shapes African swine fever virus serotype-specific locus evolution
Source: Sci Rep. 2020 Oct 28;10:18474. doi: 10.1038/s41598-020-75377-y (PMC7794389; doi:10.1038/s41598-020-75377-y)

S3. The protein alignments of ASFV C-type lectin (*EP153R*). The amino acids under the selective pressure according to the FEL analysis are marked by asterisk.

A

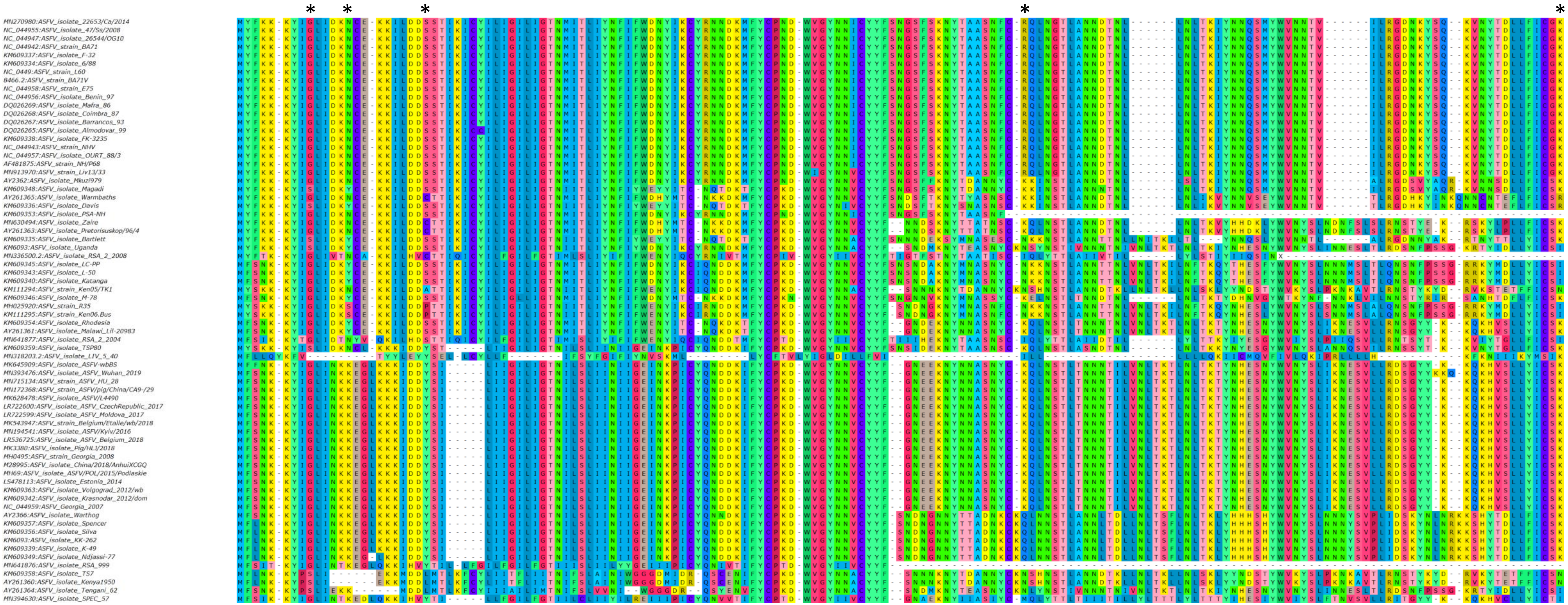

S3. The protein alignments of ASFV CD2v (*EP402R)*. The amino acids under the selective pressure according to the FEL analysis are marked by asterisk. The alignments were produced by UGENE v35.1 [52].

B

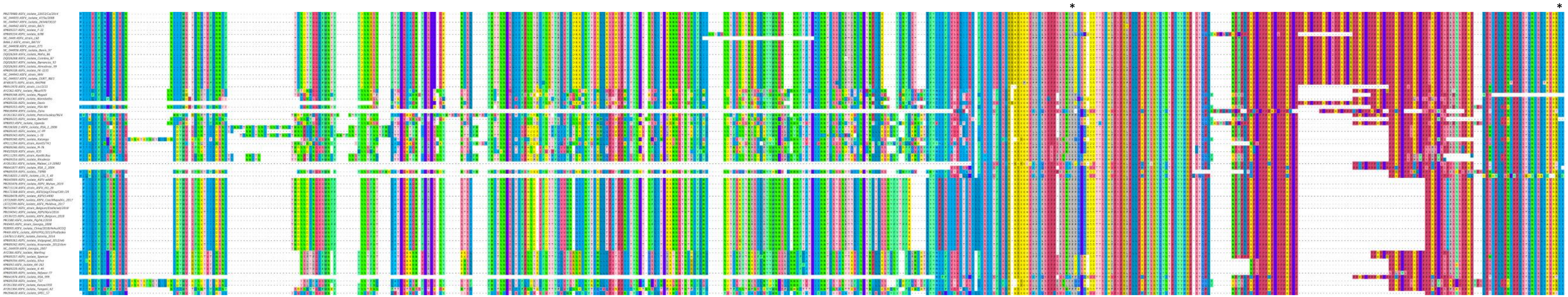

Supplement: Supplementary file 5 — Supplementary Table S3. [file 41598_2020_75377_MOESM5_ESM.pdf]
